# Primary Care SHOPping intervention for cardiovascular disease prevention (PC-SHOP): protocol for a randomised controlled trial to reduce saturated fat intake

**DOI:** 10.1136/bmjopen-2018-027035

**Published:** 2019-04-15

**Authors:** Carmen Piernas, Melina Tsiountsioura, Nerys M Astbury, Claire Madigan, Paul Aveyard, Susan A Jebb

**Affiliations:** Nuffield Department of Primary Care Health Sciences, University of Oxford, Oxford, UK

**Keywords:** Saturated fat, LDL cholesterol, grocery shopping, behavioural intervention, primary care

## Abstract

**Introduction:**

A diet high in saturated fat (SFA) increases the risk of cardiovascular disease (CVD) and intakes in the UK exceed dietary recommendations. The Primary Care Shopping Intervention for Cardiovascular Disease Prevention (PC-SHOP) study aims to test the effect of an intervention for people with raised low-density lipoprotein (LDL) cholesterol involving health professional (HP) advice alone, or in combination with personalised feedback based on nutritional analysis of grocery store loyalty card data, on SFA intake and blood lipids in comparison with no intervention.

**Methods and analysis:**

PC-SHOP is a three-arm parallel randomised controlled trial with an allocation ratio of 1:3:3 (‘no intervention’: n=16, ‘brief support’: n=48, ‘brief support plus shopping feedback’: n=48, respectively). Participants with raised LDL will be recruited from general practitioner (GP) practices for a 3-month intervention period. In brief support, an HP will deliver a behaviourally informed 10 min consultation and provide a written self-help guide to inform and motivate people to reduce their SFA intake. In brief support plus shopping feedback, the participants will receive the same HP-led behavioural support and, based on data from their grocery store loyalty card, personalised feedback on the SFA content of their grocery shopping, identifying high SFA purchases and suggesting swaps to similar but lower SFA items.

Measurements for the primary and secondary outcomes will be collected at baseline and at follow-up (3 months). The primary outcome measure will be the between-group difference in the reduction of SFA intake between baseline and follow-up. Secondary outcomes include changes in blood lipids and SFA content of food purchases, with process measures to consider the feasibility and acceptability of the intervention.

**Ethics and dissemination:**

This study has been reviewed and approved by the National Health Service Health Research Authority Research Ethics Committee (Ref: 17/SC/0168). The trial findings will be disseminated to academic and HPs through presentations at meetings and peer-reviewed journals and to the public through the media. If the intervention is effective, the results will be communicated to relevant stakeholders, including policymakers and retailers.

**Trial registration number:**

ISRCTN14279335; Pre-results.

**Strengths and limitations of this study**

- A novel intervention for people with raised low-density lipoprotein cholesterol involving health professional advice alone, or in combination with personalised feedback based on nutritional analysis of grocery store loyalty card data.
- If effective, a similar intervention could have a significant population impact and be cost-effective, even if the effect size is smaller than more intensive interventions to reduce saturated fat intake.
- Dietary intake measurements (primary outcome) come from self-reported dietary questionnaires, which could be affected by misreporting of intake.
- The effect of the intervention is measured after 3 months and may not be sustained in the longer term.

## Background

Poor diet is an important risk factor for cardiovascular disease (CVD). Saturated fat (SFA) increases the production of low-density lipoprotein (LDL) cholesterol and decreases its clearance from the body through suppression of the LDL receptor activity, which is one of the major intermediate risk factors for CVD.^1^ A Cochrane meta-analysis of randomised controlled trials (RCTs) to reduce SFA intake compared with a control group estimated a significant reduction in LDL cholesterol of 0.19 mmol/L (95% CI −0.33 to –0.05) and 17% reduction in CVD events.^2^ Meta-analyses of RCTs in which SFA has been specifically replaced by polyunsaturated fat (PUFA) have estimated a 19% reduction in CVD events,^3^ with particular improvements in vascular function attributed to increases in monounsaturated fat (MUFA).^4^ In controlled feeding trials, LDL decreased by 0.56 mmol/L when 5% of energy from SFA was replaced by PUFA.^5^

Progress in reducing SFA through public education programmes is slow, and SFA intake in the UK (13.5% total energy) and many other high-income countries remains much higher than national dietary recommendations or those from the WHO that suggest a maximum of 10% total energy. The National Institute for Health and Care Excellence in the UK recommends providing individual lifestyle advice to those at risk of CVD to achieve a meaningful reduction in SFA intake.^6^ Previous research has established that it is possible to achieve a reduction in SFA intake by providing appropriate food substitutes in place of higher SFA products, in combination with intensive and tailored counselling.^7 8^ However, systematic reviews of the impact of dietary advice and behavioural counselling to reduce SFA show only modest effects, usually confined to interventions involving specialist staff and high intensity behavioural support.^9 10^

Given that >70% of the population in the UK exceed the dietary recommendations for SFA, dietary interventions need to be suitable to be delivered at scale.^11^ We have previously shown that brief behavioural interventions delivered in routine primary care can support people who are obese to engage with a weight loss programme with the potential to achieve population-level impact.^12^ However, there is very limited evidence on similar approaches aimed at improving the quality of the diet among people at risk of CVD. A pilot trial of dietary counselling for 61 patients with raised LDL cholesterol in a primary care setting has shown a short-term beneficial effect on LDL cholesterol (−0.32 mmol/L, p<0.05 in the active intervention arm at 6 months compared with baseline),^13^ but as yet, there is no effective intervention that is sufficiently scalable and practical for routine delivery.

Food purchasing is a key antecedent of food consumption, hence improving the nutritional quality of food purchases presents a clear opportunity to intervene. Grocery shopping accounts for 71% of the weekly expenditure on food and drinks in the UK, including a large proportion of foods that are major sources of SFA in the diet such as meat, dairy, ready meals, cakes and biscuits.^14^ Individual-level interventions targeting the nutritional quality of grocery purchases could improve diet quality, especially among those motivated to change. However, previous evidence suggests that information provision alone might not be sufficient for sustained dietary change, and other behavioural strategies may be needed.^15^

Systematic reviews have identified effective intervention components for individual dietary change, including providing tailored dietary advice, information, self-monitoring and personalised feedback.^16 17^ In-store information provided at the point of purchase has been shown to improve the nutritional quality of food purchases and increase fruit and vegetable purchases.^18^ A previous intervention using an online grocery store showed a 10% reduction in SFA from food purchases by recommending lower SFA options.^19^ Loyalty card schemes operated by major grocery stores can track purchases made in the store. They are already used by retailers to provide tailored information and offers to encourage specific purchases. If purchase information was linked to a nutritional database, it is plausible that they could provide nutritional feedback to support healthier food purchases.

### Study aims

The Primary Care Shopping Intervention for Cardiovascular Disease Prevention (PC-SHOP) study aims to develop and test a behavioural intervention to promote reductions in SFA intake among patients in primary care with raised LDL cholesterol who are willing to try to change their diet. The hypothesis is that an intervention involving health professional (HP) advice alone can motivate people to reduce their SFA intake (primary outcome) and lower LDL cholesterol (secondary outcome) compared with no intervention and that providing additional, personalised feedback based on the nutritional content of grocery purchases will be more effective than brief advice only or no intervention.

Other secondary outcomes include changes in other blood lipids and the SFA content of food purchases, with process measures to consider the feasibility and acceptability of this novel intervention.

## Methods

### Trial design

The PC-SHOP study is a randomised, three-arm parallel controlled open-label trial with blinded assessment of the primary outcome. Participants will be individually randomised to either the control group or one of two active interventions for 3 months. The primary outcome measure will be the between-group difference in the reduction of SFA intake between baseline and 3-month follow-up. Recruitment of participants started the 21 March 2018 and is expected to close by 10 October 2018. Follow-up is expected to be completed by 16 January 2019.

### Trial setting and recruitment

Recruitment of participants will be performed through primary care practices in Oxfordshire, UK. Around four practices will be identified to take part through the clinical research network. GP practices that are located near the participating grocery store (which provides loyalty card data for this study) will be purposely invited to ensure that potential participants shop usually at the collaborating grocery store.

Participating GP practices will search their records for eligible participants who meet the essential inclusion criteria: (A) adults and (B) with a medical record of high LDL cholesterol (>3.5 mmol/L) or high total cholesterol (>5.5 mmol/L) over the previous 5 years. The GP will mail a letter inviting the person to contact the study team if they are interested in taking part or seeking further information.

### Inclusion criteria

- Aged ≥18 years.
- LDL cholesterol ≥3 mmol/L as measured during recruitment.
- Willing to make changes to their diet in order to reduce CVD risk.
- Have responsibility for the majority of the household food/grocery shopping (complete at least half of their household shopping).
- Shopping at the collaborating grocery store (at least every 2 weeks in store and/or online).
- Have a loyalty card registered exclusively under their name before recruitment.
- Access and ability to use a computer with internet connection.
- Willing and able to give informed consent for participation in the study.

### Exclusion criteria

- Unable to read and understand the instructions provided in English.
- Self-reported pregnancy or planning to become pregnant during the course of the study.
- Started cholesterol-lowering medication (eg, statins) in the last 3 months.
- Planned changes to cholesterol-lowering medication in the next 3 months.
- Existing cardiovascular conditions: heart attack, stroke or new diagnosis of atrial fibrillation within the last 3 months; heart failure of grade II New York Heart Association; and more severe or prolonged QT syndrome, angina, arrhythmia or familial hyperlipidaemia.
- Currently or recently (within the last 3 months) participating in another intervention study that likely affects the outcomes measured in this study.
- Participants GP judges them unsuitable for the study

### Participant flow

**Figure 1:**
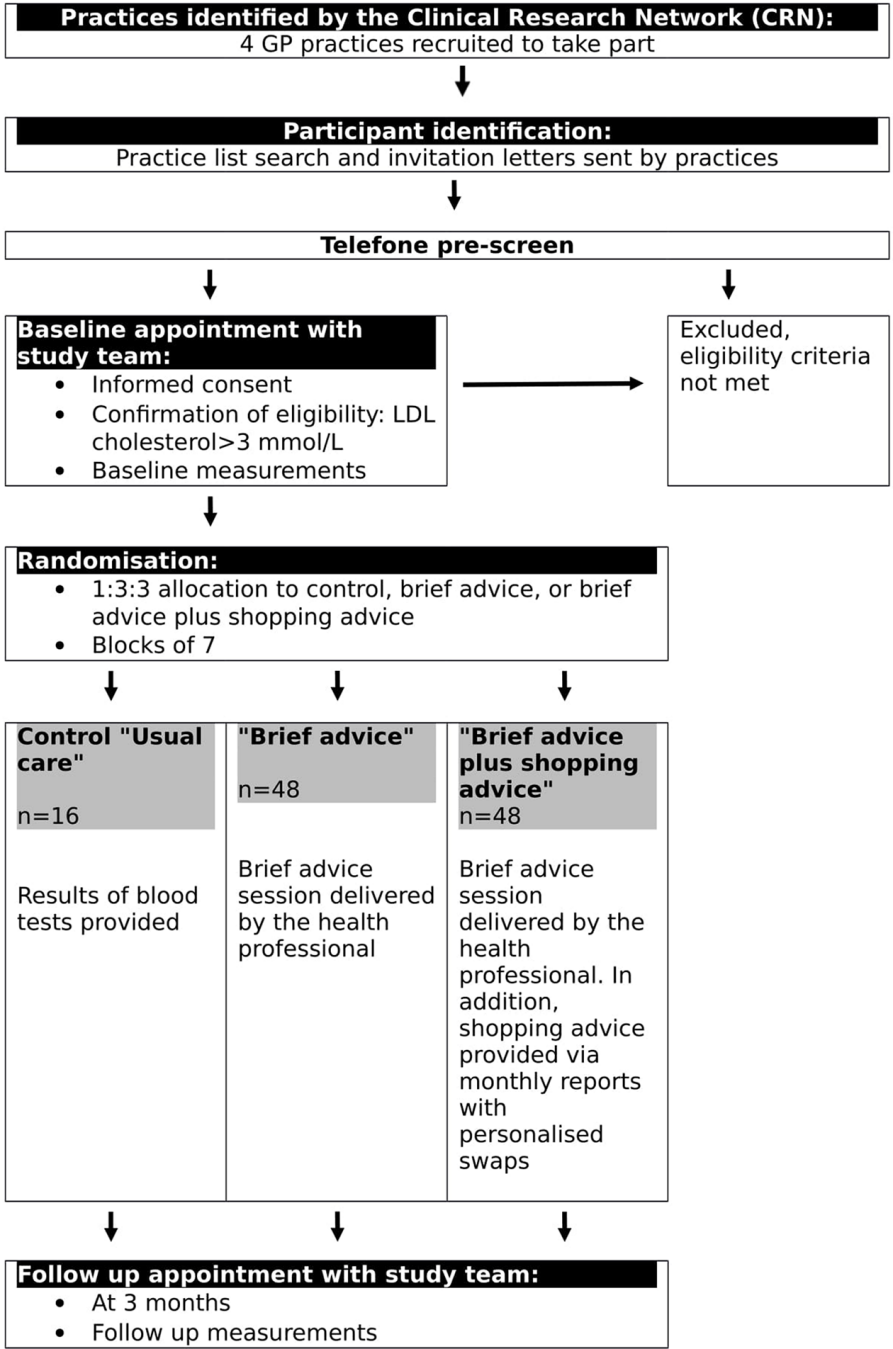
Participant flow. GP, general practitioner; LDL, low-density lipoprotein.

Interested candidates who contact the central research team will be assessed over the phone to check eligibility (figure 1). Those who meet all the eligibility criteria will be scheduled for a baseline appointment. Candidates will be asked that prior to baseline visit to complete a 24-hour dietary recall (using the Oxford WebQ, a validated online questionnaire performed on a computer.^20^

At the baseline visit, participants will provide full informed consent before completing a second online 24-hour dietary recall, and the researcher collected measures of weight, height, blood pressure, demographics, relevant medical history and grocery store loyalty card number (online supplementary appendix 1). A fasting capillary blood sample will be analysed using a point of care device (Alere Cholestech LDX) to determine total cholesterol as well as high-density lipoprotein (HDL), LDL and triglycerides. If the blood tests confirm that the participants have LDL cholesterol ≥3.0 mmol/L and they have completed two web based dietary recalls, they will be randomised to one of the three intervention groups. Data will be recorded in a web-based data capture system (RedCap), which is hosted by the Primary Care Clinical Trials Unit of the University of Oxford.

10.1136/bmjopen-2018-027035.supp1 Supplementary data

Soon after the baseline visit and once participants have been randomised, HPs from participating GP practices will contact each participant to book an appointment to receive their intervention (to those randomised to receive intervention). HP will be encouraged to make contact as soon as possible to book this appointment.

#### Follow-up

All participants will meet with the research team at 3 months for a follow-up visit, in which some outcome measures collected at baseline will be repeated, including the blood sample, the 24-hour dietary recalls, weight, blood pressure and a questionnaire to assess acceptability of the intervention.

### Sample size

Highly controlled intervention studies in populations with increased risk of CVD have achieved absolute changes in %SFA intakes of around 4%–5%,^7 8^ while a low-intensity primary care-based intervention showed a short-term change in LDL cholesterol of 0.32 mmol/L.^13^ We judged a 3% difference in SFA reduction to be realistic goal to provide clear evidence of a clinically meaningful intervention. Attrition rates have been reported to be low in short-term primary care-based trials,^13 21^ and we assumed a loss to follow-up of 10%. In order to detect a difference in the reduction of saturated fat intake (%SFA) between baseline and follow-up of 3% (3% SD) between each intervention and control with 90% power and two-sided α=0.05 using intention-to-treat analysis will require 16 participants in the control group and 48 in each intervention arm, including 10% attrition and statistical adjustment for multiple comparisons in the primary outcome. By recruiting 48 participants in each active intervention arms, we will be able to detect a further difference between these two arms of 2% (3% SD) with 90% power. This sample size will achieve 60% power to detect a change in LDL cholesterol of 0.3 mmol/L (SD 0.8) between each intervention and control using a two-sided α=0.05. A total number of 112 participants will be recruited for this study, with 16 participants allocated to control and 48 participants allocated to each of the two active interventions.

### Randomisation

Participants will be individually randomised in a 1:3:3 ratio, to either the control group or one of two active interventions. A computer-generated randomisation sequence will be generated by an independent statistician using randomly varying block sizes of 7. The allocation will be revealed to the recruiting researcher using an online programme (RedCap).

### Blinding

Provision of dietary advice and the preparation of personalised shopping reports for participants means that it will not be possible to blind the participants or the research team to the treatment allocation. The primary outcome will be a measure of SFA intake collected through a web-based questionnaire that individuals will complete individually without any involvement from the study team and is assessed blind by an independent outcome assessor. The brief advice intervention will be delivered by the practice nurse who is not aware of the treatment allocation within the active intervention arms.

### Interventions

#### Theoretical framework

The two active interventions will incorporate strategies that use the Behaviour Change Wheel COM-B model as a theoretical framework to guide the choice of behavioural strategies.^16 22^ Briefly, the proposed interventions aim to allow participants to develop and sustain the capability, opportunity and motivation to make changes to diet (table 1).^16^ Psychological capability will be enhanced by educating participants on SFA and health risks as well as information on the most appropriate swaps to decrease their SFA intake. Motivation will be promoted by the HP advice that will provide further stimulus to attempt the change and facilitate understanding of the value of these changes on health. Opportunity will only be supported by the provision of the shopping advice that will highlight the top three individual sources of SFA and proposed swaps to those, therefore making healthier choices easier to spot by participants. This shopping advice will also provide enhanced motivation by allowing participants to track and monitor their progress over time.

**Table 1:** Summary of behaviour change intervention components, targeted determinants and behaviour change techniques used in the PC-SHOP study following the Behaviour Change Wheel framework^22^

Intervention components	Primary message or resource	Targeted determinant	Intervention functions and linked behaviour change techniques
1. Brief advice session delivered by a practice nurse			
1.1. Discussion of CVD risk and motivational advice from nurse	Importance of reduction of LDL cholesterol for CVD prevention. Importance of SFA reduction to reduce LDL cholesterol.	Knowledge. Health values.	Education Information about health consequences. Persuasion Credible source (nurse). Information about health consequences.
1.2. British Heart Foundation leaflet	Importance of SFA reduction to reduce LDL cholesterol. Importance of different types of fat for health. Label reading. Planning and shopping lower SFA foods. Trying new cooking methods. Encourage meat-free day. Portion sizing and measuring.	Knowledge. Health values. Skills. Self-efficacy.	Education Information about health consequences. Persuasion Credible source (BHF). Information about health consequences. Training Instruction on how to perform behaviour.
2. Personalised feedback on food shopping			
2.1. Summary of % SFA in previous food purchases and top three foods contributing to SFA	Contribution to SFA to the overall quality of the basket. Importance of decreasing SFA for health. Contribution of specific foods to total SFA in the basket. Importance of specific types of foods for SFA.	Knowledge. Health values. Skills. Self-efficacy. Behavioural regulation.	Education Feedback on behaviour. Feedback on outcome of behaviour. Self-monitoring of behaviour. Self-monitoring of outcome of behaviour. Persuasion Feedback on behaviour. Feedback on outcome of behaviour. Incentivisation Feedback on behaviour. Feedback on outcome of behaviour.
2.2. Suggested swaps to top three foods	Encourage lower SFA options. Trying new products with lower SFA. Magnitude of reduction in total SFA.	Knowledge. Skills. Self-efficacy. Environmental context.	Education Prompts/cues. Training Instruction on how to perform behaviour, Enablement Prompts/cues. Action planning.

BHF, British Heart Foundation; CVD, cardiovascular disease; LDL, low-density lipoprotein; PC-SHOP, Primary Care Shopping Intervention for Cardiovascular Disease Prevention; SFA, saturated fat.

#### ‘Brief support’ group

Participants allocated to the brief support intervention will attend a single one-to-one appointment with an HP, usually a nurse or a healthcare assistant, who have received specific training in delivering the intervention. During the 10 min appointment participants will be provided information on the importance of dietary change to reduce CVD risk and advice motivation to reduce SFA based on the British Heart Foundation ‘*Cut the Saturated Fat*’ chart^23 24^ and information from the National Health Service Choices website (provided in printed form). This session aims to inform participants about the benefits of SFA reduction and encourage them to attempt it. Dietary advice will be particularly focused on explaining the different sources of fat and the most appropriate changes proposed in the BHF guidance to decrease SFA, primarily by substitution with lower SFA options (eg, change from regular beef to lean beef) or to substitute SFA with monounsaturated fat and PUFA sources (eg, change from butter to lower fat vegetable spread).

Practice nurses and healthcare assistants will be trained for around 45 min to deliver standardised sessions that will include two principal discussion points. First, participants will be provided with the results of their blood tests and will be informed about the different types of blood cholesterol and its importance for CVD risk. They will highlight that diet is an important modifiable risk factor that can potentially help them decrease their CVD risk by lowering LDL cholesterol. The second point of discussion will focus on the major sources of SFA in the diet for the population at large, including dairy, meat and cakes/biscuits. Participants will be given the opportunity to ask any questions with regards to the information provided, and the HP will finish the session by emphasising that the proposed changes can achieve results in a short time period.

At the final follow-up visit after 3 months, the study team will provide participants allocated to the brief support group with a report on the nutritional composition of their food purchases over the preceding 3 months.

#### ‘Brief support plus shopping feedback’ group

In addition to the brief advice session detailed above, participants allocated to the brief support and shopping feedback group will also receive a personalised report based on their previous shopping. The first report given at baseline will summarise the previous 3 months, while the subsequent monthly reports will summarise each month during the intervention period. These reports will be sent via email and post by the research team.

The shopping report will include information on the mean weekly SFA content of their food shopping and will identify the major contributors to SFA, together with suggestions for one-for-one swaps for foods containing less SFA but with similar functional characteristics, for example, an alternative fat spread or dessert with less SFA. Participants will be encouraged to use the monthly reports to monitor their progress in reducing SFA in their grocery purchases (online supplementary appendix 2).

#### ‘No intervention’ group

Participants in the control group (‘no intervention’) will be informed of the results of their blood tests by the study team. They will receive no further intervention at this time. At the final follow-up visit 3 months later, the study team will provide a copy of the BHF booklet and a shopping report on the nutritional composition of their food purchases over the preceding 3 months.

#### Generation of shopping reports for the intervention

A novel aspect of this intervention is the personalised shopping report. Transaction data will be obtained from the loyalty card system and will be received weekly from the collaborating grocery store for each participant in the trial. Transaction data will contain the bar code of each product bought, the number of units and the date each product was bought. Only those randomised to the shopping advice group will receive a report each month with details of their purchases over the previous weeks. Participants randomised to the brief support or control groups will only receive a shopping report at the end of the study.

We will use a database of approximately 20 000 food and drink products that includes own-brand and branded products with their nutrient information per 100 g and per serving as found in the nutrient information panel, as well as information on brand, product description, ingredient list, bar codes, volume and base price. This database contains the amount of SFA for each product. Using this database, we have developed a software programme that has an inbuilt algorithm to calculate the nutritional composition of the purchased groceries, identifies the five main contributors to SFA and suggests alternatives to these products which have lower SFA content. The first step of the process is to enter the list of purchased products obtained from loyalty card data over a specified period to the programme. In the second step, the programme automatically calculates the amount of SFA in the total shopping and the average per week over the specified period before identifying the five products that contribute most to the SFA content of the purchases. It then generates a lower SFA ‘swap’ for each of these five products. The swaps are generated using an algorithm that identifies all products with similar product size to the original and within the same functional food category. All matching products are ranked by ascending SFA content. The product chosen as the ‘swap’ is the product at the top of this list. The information is then used to generate an individual report of (online supplementary appendix 2). During feasibility testing of this novel intervention, all reports are individually checked by a researcher for accuracy of the nutritional data and credibility of the swap, which may necessitate changes to the initial categorisation of foods.

### Outcomes

#### Primary and secondary outcomes

The primary outcome measure will be the between-group difference in the change in saturated fat intake (%SFA) between baseline and follow-up, measured using 2×24-hour dietary recalls using the Oxford WebQ questionnaire.

##### Secondary outcomes

- Between-group differences in the change in SFA from total purchases between baseline and follow-up and in the proportion of food items with high/low SFA in the final basket (eg, products with ≥/≤1.5 g SFA/100 g).
- Between-group differences in changes in LDL cholesterol, HDL cholesterol, total cholesterol, non-HDL cholesterol, total/HDL ratio and triglycerides between baseline and follow-up.
- Between-group differences in changes in the intake of SFA (kcal), total fat (%EI, kcal), poly- and monounsaturated fats (%EI, kcal) and high/low SFA foods between baseline and follow-up using 2×24-hour dietary recalls.

##### Non-efficacy outcomes

- Between-group differences in changes in energy intake, total sugars and fibre using 2×24-hour dietary recalls.
- Between-group differences in changes in energy density, total fat, sugar, fibre and salt from purchases and in the total cost of the shopping basket (£).
- Between-group differences in changes in systolic and diastolic blood pressure.
- Between-group differences in changes (absolute [kg] and relative [%]) in body weight.

#### Feasibility and process evaluation measures

- Recruitment rate: willingness of participants to take part in the study and be randomised. This will be measured using the number of participants who accept the invitation, consent to take part in the study and are randomised at baseline.
- Follow-up rate: programme attendance and retention, calculates as the number of participants who return for the follow-up visit at 3 months.
- Acceptability of the intervention for participants and HPs; knowledge about SFA and health, motivation and swaps accepted. This will be measured using questionnaires at baseline and 3 months; and also using the number and types of swaps purchased during the intervention.
- Fidelity of the interventions: participants will be asked to consent to have the HP session audio-recorded to allow for fidelity testing. The purpose of fidelity testing is to determine: (A) if the brief advice session in being delivered according to the intended protocol; (B) to assess the number, frequency, length and content of the sessions within each group.

#### Qualitative study

Once the main study has finished follow-up, we aim to conduct a semistructured, one-to-one, 30 min telephone interview with a subsample of the PC-SHOP study participants to explore issues related to food purchasing behaviours, including knowledge, perceived barriers, facilitators and actions, contextual influences and value of the healthcare advice provided. This qualitative study will play a valuable role as part of the process evaluation of the trial and will help in the interpretation of the findings of the trial. If the trial outcome is promising, it may point to areas that could be enhanced ahead of any future definitive trial to test the effectiveness of the intervention to reduce LDL cholesterol.

Verbatim transcriptions will be analysed using the NVivo 11 software program. A thematic analysis approach will draw out themes, categories and nodes from the data pertaining to the: knowledge, perceived barriers and facilitators, contextual influences and value of the healthcare advice provided. One researcher will deductively code the transcript data against an initial thematic framework using the Framework Method, which comprises five steps: familiarisation, developing a thematic framework, indexing, charting, mapping and interpretation.

This qualitative study will include participants from the brief advice plus shopping advice intervention arm who are willing to be interviewed. We will continue to recruit until sufficient numbers and data saturation are achieved, estimated based on experience to be approximately 28 people.

### Planned analyses

A statistical analysis plan has been published in the ISRCTN14279335 (11 March 2019) prior to trial end date and data analysis.

The primary statistical analysis of efficacy outcomes will be carried out on the basis of intention to treat. We will endeavour to obtain full follow-up data on every participant, but we will inevitably experience the problem of missing data due to withdrawal, loss to follow-up or non-response to questionnaire items.

We will analyse the primary and secondary outcomes with a linear regression model with adjustment for practice and baseline values. We will assess the sensitivity of the analysis to different assumptions about missing data using two imputation methods commonly used: completer only analysis and baseline observation carried forward.

The primary analysis will test for:

- A difference in the change (from baseline to follow-up) in % SFA intake between each intervention group compared with control.
- A difference in the change (from baseline to follow-up) in % SFA intake between the two active intervention arms.

The results from the trial will be prepared as comparative summary statistics (difference in means) with 95% CIs. All the tests will be done at a 5% two-sided significance level.

Using the appropriate interaction terms in the above-mentioned models, we will perform subgroup analyses of the primary and secondary outcomes by socioeconomic characteristics (SES), as a previous study has shown that households from a lower socioeconomic status purchase a higher proportion of energy from less healthy foods with smaller but important differences in the SFA content from purchased foods that may lead to differential effectiveness.^25^ While the study is not powered to look for such effects here, this information will inform planning of subsequent research.

Acceptability of the intervention by the participants will be collected by questionnaire and will be summarised by presenting the frequencies of each response.

### Patient and public involvement (PPI)

At the funding application stage of this study, we sought detailed and specific feedback on our research plans from 32 PPI members of our department panel, who all have a least one feature of the metabolic syndrome. We observed that more than half (63%) had never been advised by a healthcare professional to cut SFA intake in order to reduce their CVD risk. However, all except two have tried to do so. While the majority of them felt quite confident that they knew the major sources of SFA in their diets, all of them thought that receiving feedback on the SFA content of their baskets would be helpful or very helpful. Most of them also reported using a loyalty card and 72% thought that it would be a good idea to collect and use the information on their loyalty card. Nine of them (28%) were not sure about it or had mixed feelings about the use of these data.

At the initial stages of the study, we also established a small advisory panel comprising 13 PPI members. This group has informed all stages of the project, including research and intervention design (eg, reviewing all the materials given to participants during the intervention), methodology for obtaining purchasing data and the format and manner of communication to participants. Future involvement includes plans to advise on the preliminary dissemination (eg, helping develop an infographic leaflet to communicate the final results to the wider population and the study participants).

### Study dissemination

The trial findings will be disseminated to academic and HPs through presentations at meetings and peer-reviewed journals and to the public through the media. Participants will be sent a summary of the study findings at the time when the main article is published. If the intervention is effective, the results will be communicated to relevant stakeholders, including policymakers and retailers.

## Discussion

The PC-SHOP study will test whether an intervention involving HP advice alone or in combination with personalised feedback and monitoring of food purchases is more effective than usual care in reducing the intake of SFA, the SFA content of food purchases and improving LDL cholesterol concentrations, a biomarker of CVD risk.

One intervention group will receive a brief advice session from the healthcare professional that will provide motivation to attempt dietary change by highlighting the importance of dietary SFA and the impact any reduction could make to LDL cholesterol. However, recognising that HPs may lack sufficient time and training to go beyond general messages about how to reduce SFA, we also propose a behaviourally enhanced intervention to provide personalised feedback on shopping to aid self-monitoring of dietary habits, with the potential to further support and sustain behaviour change.^17^ Accordingly, the second intervention group will receive the same brief advice session in combination with food purchasing advice and lower SFA swap suggestions to provide a more complex theoretically informed and behaviourally enhanced intervention to boost the participants’ capability, opportunity and motivation to make dietary changes. By drawing on the power of data already collected by grocery stores, we will be able to automate the process to deliver personalised advice that would otherwise require considerable time from specialist practitioners, usually dietitians. The outcomes from these two interventions will be compared with usual care (no intervention), in which a control group will receive their blood test results without any further advice.

Here we will examine the effect of the intervention on SFA intake as the primary outcome and, if promising, we will plan for a definitive trial to test the effectiveness of the intervention to reduce LDL cholesterol over 1 year. The main limitation of this study is that the dietary intake measurements come from self-reported dietary questionnaires that could be affected by misreporting of intake. Also, we will measure the effect of the intervention after 3 months, which is a relatively short period of time, and may not be sustained in the longer term. Major strengths include the novelty and low cost nature of the proposed intervention as well as the potential to be automated to be rolled out to reach large numbers of people who could benefit from this type of advice. If found to be effective, it could have a significant population impact and be very cost-effective, even if the effect size is smaller than more intensive interventions to reduce SFA intake.

This study will also be the first test of a novel programme to analyse information from loyalty cards and provide dietary advice, allowing us to refine the algorithms and learn lessons for future developments of relevance both to researchers and to retail partners who may wish to offer this type of support to their customers. Process measures from the trial will provide insights into the acceptability to participants and HPs of both brief advice and shopping advice and the impact on nutritional knowledge and motivation to change. It will also address key uncertainties for any future trials focused on dietary change in primary care, particularly in this group of patients, for example, the feasibility of recruiting patients who are willing to change their diet and follow-up rates.

If the brief advice is successful in reducing SFA intake and/or LDL cholesterol, the training programme and patient resources can be rapidly disseminated for others to use. If the shopping report is acceptable and effective, it could provide a tangible opportunity for grocery stores to offer such a service and to play a more proactive role in shaping healthier choices for customers trying to achieve a healthier diet.
