# Supplementary material for: Primary Care SHOPping intervention for cardiovascular disease prevention (PC-SHOP): protocol for a randomised controlled trial to reduce saturated fat intake
Source: BMJ Open. 2019 Apr 15;9(4):e027035. doi: 10.1136/bmjopen-2018-027035 (PMC6500228; doi:10.1136/bmjopen-2018-027035)
Supplement: Supplementary data [file bmjopen-2018-027035supp001.pdf]

Supplementary Appendix 1. Schedule of study procedures

	Baseline visit 1	Baseline visit 2	Month 3 visit
**Length of visit**	60 min	15 min	45 min
**Who conducts**	Central research team	Health professional	Central research team
**Procedures**			
Informed consent	√		
Eligibility assessment	√		
Demographics/shopping behaviours	√		√
Medical history	√		√
Weight and height	√		√
Blood pressure	√		√
Blood sample	√		√
Medication review	√		√
2 x 24h dietary recall questionnaires	√		√
Retailer loyalty card number	√		√
Questionnaires about the intervention	√		√
Randomisation	√		
Intervention delivery		√	

Supplementary Appendix 2. Example of a shopping report for the PCSHOP participants

Dear Participant,

We have analysed your shopping over the past weeks. Your shopping basket contained:

220g
saturated fat per week

We have identified the products you buy that contain the most saturated fat. We have suggested some swaps for you to make to help reduce the saturated fat in your next basket.

Why not give these a try?

Lowering your saturated fat intake can reduce your cholesterol levels and decrease your risk of heart disease and stroke.

Please note:
These numbers represent only the amount of saturated fat in your total shopping basket and are not an estimate of the amount of saturated fat you have actually eaten. They should not be compared to recommended daily dietary intakes.

Report on your purchases from 19/04/2018 to 19/07/2018

SHOP Preventing Cardiovascular disease In primary care

Try swapping these products:

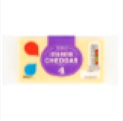	Own Label Extra Mature Cheese 800g	21.7g	**7.9g less** → saturated fat per 100g	13.8g	Own Label Healthy Extra Mature Cheese 400-450 450g	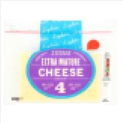
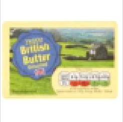	Own Label Butter Unsalted Block 250g	52.1g	**16.5g less** → saturated fat per 100g	35.6g	Willow Original Block 250g	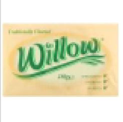
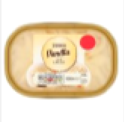	Own Label Vanilla Ice Cream 900-1000ml 900ml	7.9g	**7.1g less** → saturated fat per 100g	0.8g	Halo Top Vanilla Low Sugar Ice Cream 473ml	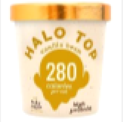
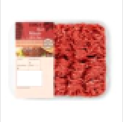	Own Label 15% Fat Beef Mince 500g	6.5g	**4.5g less** → saturated fat per 100g	2.0g	Own Label Extra Lean Steak Mince 750g	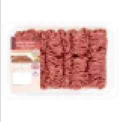
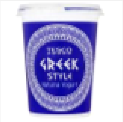	Own Label Greek Style Natural Yogurt 450-500 500g	6.3g	**6.3g less** → saturated fat per 100g	0.0g	Fage Total 0 Percent Fat Free Greek 500g	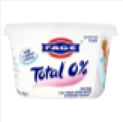

UNIVERSITY OF OXFORD
NUFFIELD DEPARTMENT OF PRIMARY CARE HEALTH SCIENCES
